# Experimental and Numerical Study of Combined High and Low Cycle Fatigue Performance of Low Alloy Steel and Engineering Application

**DOI:** 10.3390/ma14123395

**Published:** 2021-06-18

**Authors:** Zhanzhan Tang, Zheng Chen, Zhixiang He, Xiaomei Hu, Hanyang Xue, Hanqing Zhuge

**Affiliations:** 1 Department of Civil Engineering, College of Civil Science and Engineering, Yangzhou University, Yangzhou 225127, China; MX120190464@yzu.edu.cn (Z.C.); MZ120190743@yzu.edu.cn (Z.H.); MX120200577@yzu.edu.cn (X.H.); DX120200087@yzu.edu.cn (H.X.); 2 Department of Applied Mechanics and Structures, Institute of Theoretical and Applied Mechanics, Czech Academy of Sciences, 190 00 Prague, Czech Republic; 3 Department of Civil Engineering, College of Civil Engineering and Architecture, Zhejiang University of Science & Technology, Hangzhou 310023, China; 120043@zust.edu.cn

**Keywords:** low alloy steel, material experiment, combined high and low cycle fatigue, high cycle fatigue, low cycle fatigue, structural performance

## Abstract

The fatigue behaviors of metals are different under different in-service environment and loading conditions. This study was devoted to investigating the combined effects of high and low cycle fatigue loads on the performance of the low alloy steel Q345. Three kinds of experiments were carried out, including the pure high cycle fatigue (HCF) tests, the pure low cycle fatigue (LCF) tests, and the combined high and low cycle fatigue (HLCF) tests. The prediction formulae were proposed for the combined high and low cycle fatigue failure. Scanning electron microscopy (SEM) and stereo microscope were used to analyze the microstructure and fracture morphology due to different fatigue loads. Case study on the combined high and low cycle fatigue damage of a steel arch bridge was carried out based on the FE method and the proposed formula. The results show that the LCF life decreases evidently due to the prior HCF damages. The HLCF fracture surface is relatively flat near the crack initiation side, and rugged at the other half part. The fatigue damages at the bridge joints increase significantly with consideration of the pre-fatigue damages caused by traffic load. In the 100th anniversary of service, the fatigue damage index without considering the HCF pre-damage is only about 50% of the coupled damage value.

## 1. Introduction

Low alloy steels are widely used in civil or mechanical engineering fields due to the excellent material properties—e.g., the high strength, high toughness, good ductility and excellent welding property. Fatigue is one of the main degradation processes that affect the structural safety. On the one hand, the materials experience long-term high cycle fatigue (HCF) loads during the service life, such as the wind and traffic loads applied on a steel bridge or the vibration of a machinery part. On the other hand, the materials may experience cyclic loads with very large amplitude to the plastic range during an extreme event, such as seismic events [1,2,3]. Therefore, the combined effects of the high and low cycle fatigue (HLCF) performance of the low alloy steels should be carefully investigated in order to ensure the structural safety.

The high cycle fatigue performances of different kinds of steels under different service environments or load conditions have been extensively investigated. Xin et al. [4,5] adopted the Walker equation to predict the fatigue crack propagation rate of S355 and S690 grade steels at room temperature. Zhao et al. [6] studied the high cycle fatigue performance of the ferritic-pearlitic medium-carbon forging steels by rotating bending fatigue tests. Soyama et al. [7] studied the high cycle fatigue properties of the stainless-steel samples by a displacement controlled plane bending fatigue test. Makino et al. [8] studied the fatigue properties of the cast steel used in the railway bogie frame constructions and proposed a corresponding criterion for the fatigue design. Hu et al. [9] investigated the high cycle fatigue behavior of low alloy steel Q345B by the high-frequency tension and compression material tests, in which the surface temperature was monitored and analyzed. Liao et al. [10] conducted a series of strain-controlled fatigue tests of low alloy steel Q370qE at a wide range of temperatures between −60 °C and 20 °C. Adasooriya et al. [11] and Guo et al. [12] proposed a fatigue life prediction formula (S-N curve) for the structural steels exposed to corrosive environment. Electrically accelerated corrosion and axial fatigue tests were adopted by Ouyang et al. [13] to investigate the degeneration features of the fatigue life of the corroded steel bars. Wang et al. [14] established a fracture model using the short-term monitoring data to consider the coupling effect of fatigue and corrosion of an old steel bridge. Mohtadi-Bonab et al. [15] analyzed the microstructure of API X65 pipeline steel, and studied the role of inclusions in the fatigue fracture process. Chang et al. [16] studied the effect of virtual radius on the fatigue performance of alloy materials, and proposed an optimal virtual radius values suitable for thin-plate alloy welded structures. Park et al. [17] compared the difference of the fatigue behavior between the conventional steel and the fatigue-resistant steel, and they proposed a modified fatigue design curve for the application of the fatigue-resistant steel. Above-mentioned studies have revealed the fatigue properties of the steel metals under high cycle fatigue loads. Effective high cycle fatigue prediction formulae have been proposed and validated experimentally, which can be used to evaluate the HCF failure of the material during service.

In the Northridge earthquake in 1994 and the Great Hanshin earthquake in 1995, many steel bridge piers and steel buildings failed due to the low cycle fatigue loads [1,2,3]. After that, great attentions have been paid to the low cycle fatigue performance of the low alloy structural steel and the steel structures. Hu et al. [18] studied the preliminary torsional strain on the low cycle fatigue of low alloy steel Q345B, and the parameters in the Coffin–Manson relation were obtained. Low cycle fatigue failure properties of API-5L X65 steel was studied by Fatoba et al. [19] through the material experiments, and it is found that the steel exhibits non-Masing and cyclic softening behaviors during the loading process. Feng et al. [20] studied the low cycle fatigue behavior of S550steel under different strain amplitudes with different strain ratios. A modified energy-based model was proposed to predict the fatigue life of this kind of high-strength steel. Yang et al. [21] and Shi et al. [22] investigated the low cycle fatigue properties and fracture behaviors of some low yield point steels, including the LY100, LY160, and LY225 grade steels. A wide range of strain amplitudes were used from 0.5% to 6% in the experiments, and the parameters of the Coffin–Manson relation and Kuroda model were calibrated in their study. Milani et al. [23] investigated the low cycle fatigue performance of cylindrical specimens of S355 steel subjected to cyclic torsional loads. These studies focused on the cyclic fracture behavior and the life prediction formula based on the plastic strain amplitudes. Therefore, most of them tried to develop a Coffin–Manson type relation for engineering design of the steel materials. Sakane et al. [24] studied the effect of strain multiaxiality on the cracking propagations by the tension-torsion low cycle fatigue tests, and they reported the multiaxial strain state can affect the crack propagation directions. Some researchers tried to explain the fracture mechanism based on the micro-mechanism models. Tong et al. [25] studied the low cycle fatigue behavior of beam-column joints using the continuous damage mechanics model. Kanvinde et al. [26] proposed the cyclic void growth model (CVGM) to explain the low cycle fatigue mechanism of the structural steels based on the void growth theory. In order to facilitate the application of the proposed CVGM in engineering practice, Liao et al. [27,28] and Yin et al. [29] calibrated the parameters in the micro-mechanism model for the commonly used structural steels by experiments. Li et al. [30] found that the level of the stress triaxiality has a significant impact on the damage degradation parameter, and a modified calibration method was proposed in their study. These research findings and proposed methods can be used to evaluate the fatigue failure caused by LCF loadings without considering the HCF pre-damages, namely these studies focused on the LCF properties of the damage free materials. At present, most of the previous studies either focused on the pure HCF or pure LCF behavior of the alloy steels. It can be concluded that, the HCF and LCF properties of the alloy steels still belong to two individual research topics, very few studies have addressed the coupled effects of high and low cycle fatigue properties.

Even though the combined high and low cycle fatigue behaviors of the turbine blades in aero-engines were reported in a few studies [31,32,33,34,35], the loading form is a coinstantaneous combined form of the major and minor stress cycles, and the turbine blades are usually made by titanium alloys, whose mechanical properties are quite different from the low alloy steels. Hence the present work concentrated on the combined high and low cycle fatigue performance of the low alloy steel Q345qD. Microstructures of the specimen fracture surfaces were compared and analyzed. The prediction formulae for different kinds of fatigue lives were proposed. Moreover, the proposed formulae were used for the fatigue damage prediction of an in-service steel arch bridge under the combined effects of traffic and seismic loads.

## 2. Materials and Test Methods

**Figure 1:**
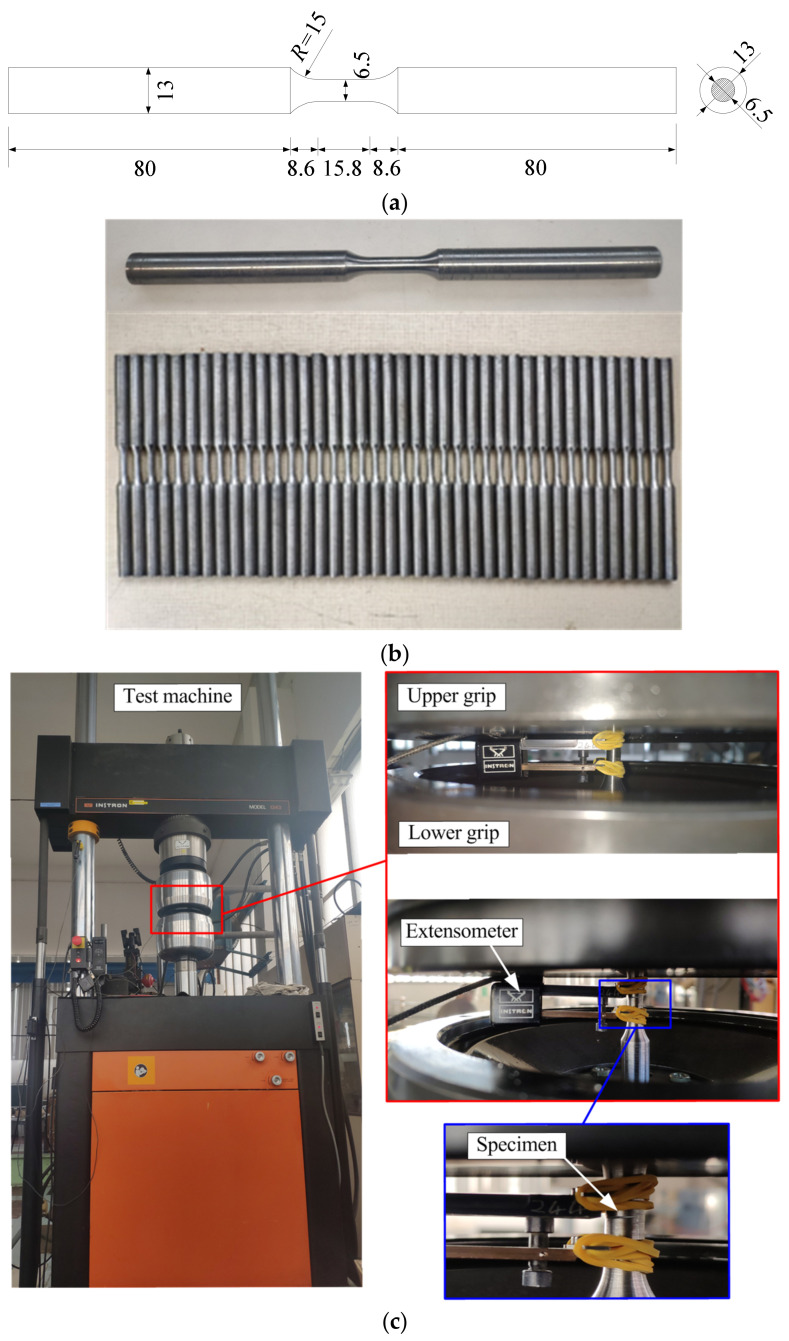
Material specimens and test setup: (**a**) shape and size of the specimen (Unit: mm); (**b**) photos of the specimens; (**c**) test machine and strain measurement.

The tested material is the low alloy structural steel Q345 (L13453) [36], which is widely used in the infrastructure construction. The chemical compositions in weight ratio of the tested material are listed in Table 1, which are provided by the steel factory (Baoshan Iron & Steel Co., Ltd., Shanghai, China). The shape and size of the specimen, as well as the test machine are shown in Figure 1. According to the specifications [37,38], the length of the specimen bar is designed as 193 mm with a parallel segment of 15.8 mm (gauge section). The diameters of the specimen at the parallel segment and the gripped end are 6.5 mm and 12.0 mm, respectively. The radius of the transition segment is 15 mm. Surface polishing was carefully carried out to remove the scratches and to ensure the accuracy of the experiment results. The tests were performed using an electro-hydraulic fatigue machine (INSTRON 1343 System 8800-ML2424) at room temperature in atmospheric conditions. The strain was measured by an extensometer of Catalogue 2620-602 at the parallel segment. The defined gauge length of the extensometer is 10 mm with a maximum measuring range of ±2.5 mm. A total of 57 specimens were tested in this study. Before the fatigue tests, the monotonic tensile tests were conducted using three specimens to get the basic mechanical properties of the low alloy steel. The average elastic modulus of the metal is 205.2 GPa, the average yield strength and the ultimate strength are 361.8 MPa and 519.7 MPa, respectively. The average plastic elongation after fracture is 28.0%.

**Figure 2:**
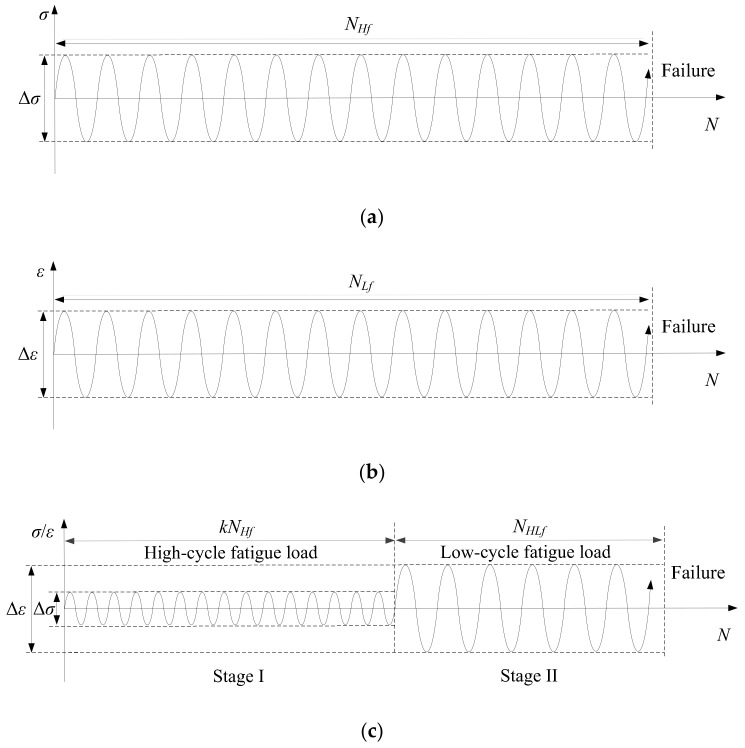
Loading methods: (**a**) HCF loading mode; (**b**) LCF loading mode; (**c**) HLCF loading mode.

The aim of the tests is to investigate the combined high and low cycle fatigue properties of the material. Therefore, three loading methods were designed as shown in Figure 2: the pure high cycle fatigue (HCF) loading mode, the pure low cycle fatigue (LCF) loading mode, and the combined high and low cycle fatigue (HLCF) loading mode. A sinusoidal waveform with a strain ratio of R = −1 was selected, and the load frequencies were set as 25 Hz and 0.033 Hz in the HCF and LCF tests, respectively [38]. The HCF loading mode was stress controlled since the stress amplitude is in the elastic range, while the LCF loading mode was strain controlled and related to large strain amplitudes that can produce plastic components. The stress control mode in the tests was actually load-controlled since the applied load amplitude can be converted into stress amplitude by the Instron machine. The HCF and LCF lives are represented as *N_Hf_* and *N_Lf_*, and Δ*σ* and Δ*ε* are the full stress and strain load amplitudes, respectively. In the HLCF tests, LCF loads were applied to the specimens with different levels of HCF pre-damages, and *k* is a parameter to describe the pre-damage level. Note that three specimens were tested in each load case to obtain reliable results by averaging the experimental data.

## 3. Test Results and Discussion

### 3.1. Test Results of High-Cycle Fatigue

High cycle fatigue tests were carried out to obtain the HCF lifetime, which can be used to determine various pre-damage levels of the specimens. Different stress amplitudes from 200 to 250 MPa were chosen to get a fatigue life less than 2 million. 12 specimens were used to determine the S–N curve for the low alloy steel. Table 2 lists the HCF test results, in which the average life of the three specimens and the coefficient of variation in the bracket are listed in the last column. The exact diameters of the gauge sections are also provided in the table.

**Figure 3:**
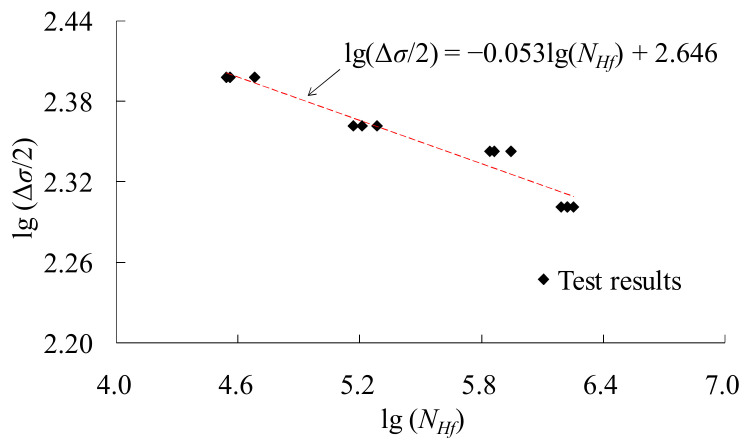
High cycle fatigue test results and the fitting equation.

Figure 3 shows the results and the fitting equation of the HCF tests. For Q345 grade steel, the constant-amplitude cyclic load between −200 and 200 MPa developed a fatigue life of 768,565 cycles. As the stress amplitude increases, the corresponding fatigue life decreases gradually. An S–N equation can be obtained according to the test results

(1) $$\mathrm{lg}\left(\Delta \sigma /2\right)=-0.053\mathrm{lg}\left(N_{Hf}\right)+2.646,\;\;\;\;R^{2}=0.936$$

### 3.2. Test Results of Low-Cycle Fatigue

The strain amplitude ranges from 1.0 to 3.0% was adopted in the LCF tests. A very low frequency of 0.033 Hz was utilized to avoid thermal effects. The test result was considered invalid if the fracture is outside of the gauge section. Fifteen specimens were used to determine the *ε*–N curve for the low alloy steel. Table 3 lists the LCF test results, in which the average life in each load case and the coefficient of variation in the bracket are listed in the last column.

**Figure 4:**
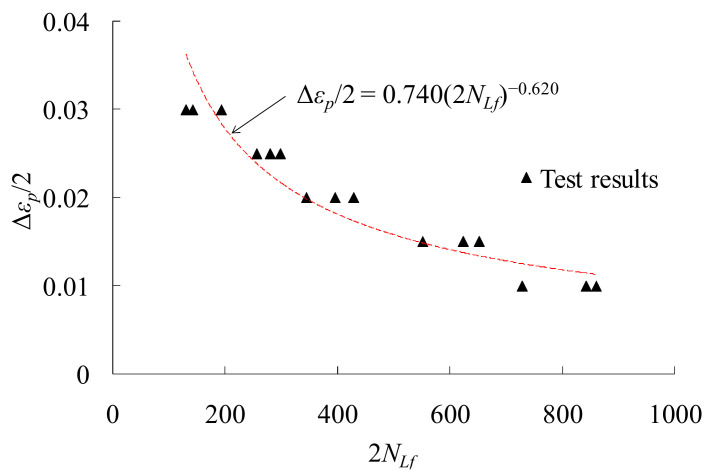
Low cycle fatigue test results and the fitting equation.

When the strain amplitude is very large, a LCF life less than 500 cycles can be obtained. In such a situation, the effect of the elastic strain component can be ignored, and the plastic strain component plays a predominant role. LCF life of the alloy steel can be predicted by Coffin–Manson formula. Figure 4 shows the results and the fitting relation of the low cycle fatigue tests. As the plastic strain amplitude increases, the corresponding fatigue life decreases gradually. The prediction formula can be obtained according to the test results

(2) $$\frac{\Delta \varepsilon_{p}}{2}=0.740{\left(2N_{Lf}\right)}^{-0.620},\;\;\;\;\;\;\;\;\;\;\;\;R^{2}=0.901$$

where Δ*ε_p_*/2 is the applied plastic strain amplitude.

The damages caused by different amplitudes of LCF load can be counted using the Miner’s rule

(3) $$D_{\mathrm{LCF}}=\sum_{i=1}\frac{n_{i}}{N_{Lfi}}$$

where *D*_LCF_ is the fatigue damage index caused by LCF load, *D*_LCF_ = 0 means the material is not damaged, while *D*_LCF_ = 1 means LCF failure occurs. *i* represents the amplitude level of the strain load, *n_i_* represents the number of the applied load cycles with the *i*-th level amplitude, and *N_Lfi_* is the fatigue life corresponding to the *i*-th level amplitude strain load.

The plastic component of the Coffin–Mansonrelation can also be expressed as

(4) $$\Delta \varepsilon_{p}N_{Lf}^{k\prime }=C$$

where *C* and *k’* are parameters related to the material property.

A cumulative damage index can be derived from Equations (3) and (4) [39]

(5) $$D_{\mathrm{LCF}}=\frac{1}{2C^{1/k\prime }}\sum_{i=1}^{n}{\left(\Delta \varepsilon_{pi}\right)}^{1/k\prime }$$

where Δ*ε_pi_* is the plastic strain amplitude in each half load loop. This equation can be used to predict the LCF damage of the alloy steel subjected to plastic strain loads with different amplitudes.

By comparison with Equation (2), the parameters can be obtained as *C* = 0.963 and *k’* = 0.620. For the low alloy steel Q345, the cumulative damage index in Equation (5) can be expressed as

(6) $$D_{\mathrm{LCF}}=0.5314\sum_{i=1}^{n}{\left(\Delta \varepsilon_{pi}\right)}^{1.6129}$$

### 3.3. Test Results of Combined High and Low Cycle Fatigue

LCF loads were applied to the specimens with different HCF pre-damage levels. Knowing the relations between the load amplitudes and the pure HCF and LCF lives, e.g., the S-N and *ε_p_*-N curves of the low alloy steel, it is possible to determine the load amplitudes in the subsequent HLCF tests. To study the effect of the HCF pre-damage on the remained LCF life, the number of the HCF load cycles was set as *k*·*N_Hf_* (*k* = 0.1–0.9) with a stress amplitude of 220 MPa in the first loading stage, while in the second load stage the strain amplitude was set as 2.5%. Thus, the HLCF test of each specimen can be completed in one day. Table 4 shows the HLCF test results, in which the average life and the coefficient of variation in the bracket are listed in the last column.

**Figure 5:**
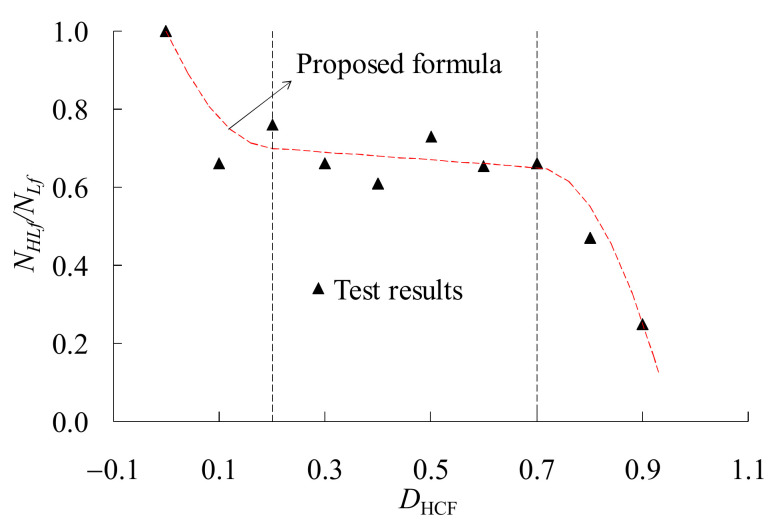
Coupled high and low cycle fatigue test results and the proposed formula.

Figure 5 shows the effect of the HCF damages on the LCF life of the material, in which *D_HCF_* (=*k*) represents the HCF damage generated in Stage I. As it can be seen, the LCF life decreases due to the preceding HCF damage. The decrease process of the LCF life can be divided into three stages. The first stage is very short with about 20% of the HCF life fraction, but the LCF life deteriorates very fast, represented by a sharp slope. The second stage is relatively long with about 50% of the HCF life fraction, and the remained LCF life deteriorates slowly. In the third stage, the LCF life drops rapidly and the HCF life fraction is about 30%. Therefore, a piecewise formula to predict the remained LCF life is proposed

(7) $$D_{\mathrm{HLCF}}=\frac{0.5314}{\gamma }\sum_{i=1}^{n}{\left(\Delta \varepsilon_{pi}\right)}^{1.6129}\;\gamma =\left\{\begin{array}{l} 7.5{\left(D_{\mathrm{HCF}}-0.2\right)}^{2}+0.70\;\;\;\;\;\;\;\;\;\;\;\;\;\;\;\;\;\;\;\;\;\;\;\;\;\;\;\;\;\;\;\;\;\;0\leq D_{\mathrm{HCF}}<\;0.2\\ -0.1D_{\mathrm{HCF}}+0.72\;\;\;\;\;\;\;\;\;\;\;\;\;\;\;\;\;\;\;\;\;\;\;\;\;\;\;\;\;\;\;\;\;\;\;\;\;\;\;\;\;\;0.2\leq \;\;D_{\mathrm{HCF}}\;<0.7\\ -10.0{\left(D_{\mathrm{HCF}}-0.7\right)}^{2}+0.65\;\;\;\;\;\;\;\;\;\;\;\;\;\;\;\;\;\;\;\;\;\;\;\;\;\;\;\;\;D_{\mathrm{HCF}}\geq 0.7 \end{array}\right.$$

where *γ* is a reduction coefficient considering the effect of the HCF pre-damage.

### 3.4. Scanning Electron Microscope Tests

**Figure 6:**
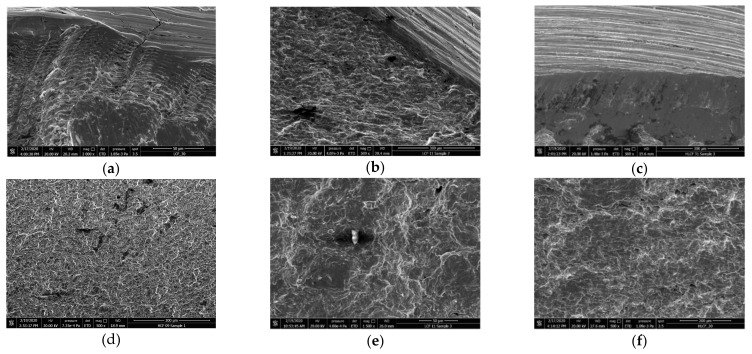
Microfractographs of the fracture surfaces: (**a**) HCF fracture near the surface; (**b**) LCF fracture near the surface; (**c**) HLCF fracture near the surface; (**d**) HCF fracture surface in the crack propagation area; (**e**) LCF fracture surface in the crack propagation area; (**f**) HLCF fracture surface in the crack propagation area.

The fracture surface was scanned using a scanning electronic microscope (SEM Quanta 450 FEG). Figure 6 shows the microfractographs of the fracture surfaces. The specimens show different fracture feathers as seen in these photographs. The HCF crack initiated from the specimen surface, where the fatigue striations can be seen. Evident dimples can be seen on the LCF fracture surface, indicating that the specimen underwent large scale plasticity before fracture. Moreover, a secondary particle is found in the LCF fracture surface, which is a sign of ductile fracture form. The HLCF fracture has the features of both the HCF and LCF fracture, such as the crack initiation from the surface and the dimples distributed on the fracture surface.

**Figure 7:**
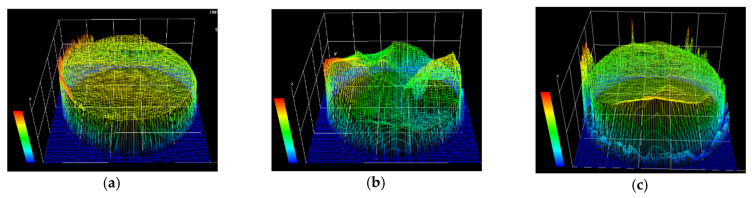
Stereograms of the fracture surface: (**a**) HCF fracture surface; (**b**) LCF fracture surface; (**c**) HLCF fracture surface.

Figure 7 shows the contours of the fracture surfaces, which were obtained by a stereo microscope (SZX-7). There are some errors of the contours along the edge of the cross-section due to the steep drop in height. The HCF fracture surface is rather flat, indicating the crack initiated from the surface and propagated approximately to the same transverse plane. However, the LCF fracture surface is rugged since multiple crack sites were existed at different heights. The HLCF fracture surface is relatively flat near the fatigue crack initiation side, while it is rugged at the other half part in the opposite direction.

## 4. Fatigue Damage Evaluation of a Steel Bridge Considering Service History

Case study of fatigue failure of an in-service steel arch bridge is studied in this section. The fatigue damages accumulated during its service life is considered before the outbreak of the seismic event. The HLCF damage is computed according to the proposed formulae in this study.

### 4.1. Structural Design and Numerical Model

**Figure 8:**
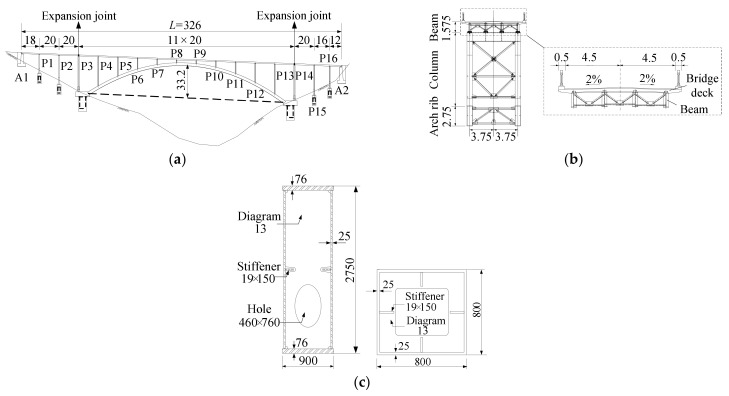
Structural design of the bridge: (**a**) elevation of the bridge (unit: m); (**b**) transverse view of the bridge (unit: m); (**c**) cross-sections of the arch rib and column (unit: mm).

Figure 8 shows the structural design of the steel bridge with a span length of 200 m, in which A1 to A2 represent the abutments, and P1 to P16 represent the columns from the beginning to the end of the structure. The rise-to-span ratio of the two arch ribs with box-section is 0.166. The dimension sizes of the cross sections of the arch ribs and the columns are 0.9 × 2.75 m and 0.8 × 0.8 m, respectively. The deck of the bridge is composed of four beams with I-type section and a concrete slab. Lateral struts are designed for the connection of the arch ribs and columns.

**Figure 9:**
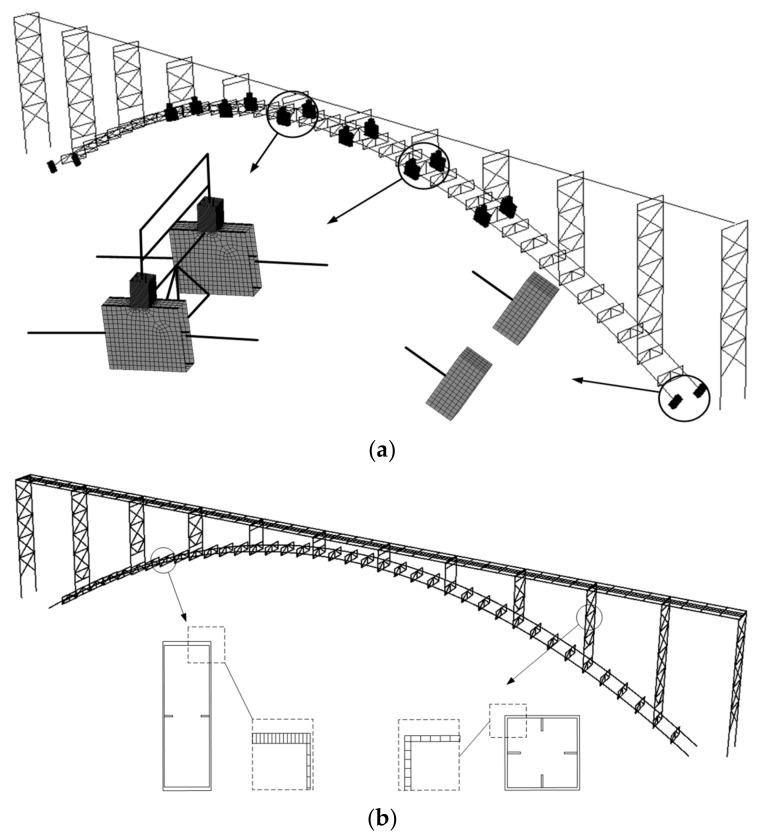
FE models: (**a**) hybrid element model; (**b**) beam element model.

Figure 9 shows the FE models of the bridge, which were created using the commercial FE software ABAQUS. Numerical analysis was performed of the structure between P3 and P14 columns since two expansion joints were set there. The dynamic analysis results show that, the arch-column joints in the mid-span position and the arch feet are prone to seismic damages under the excitation of a strong earthquake. Therefore, in the hybrid element model, these joints were modeled by fine shell elements in a smaller size, while the other parts of the structure were modeled using fiber-beam elements [40]. The sectional fibers were set as 120 and 80 in number for the arch rib and the column, respectively. For comparison purpose, a full fiber-beam element model was also created. Fixed boundary conditions were used at the bottom of columns P3 and P14, and the arch rib feet. Shell elements of S4R and beam elements of B31 were adopted for the numerical analysis. The Chaboche combined hardening model of the steel material was adopted, which can take both the isotropic and kinematic hardening effects into account [41]. The material properties were verified by the experiment in the present study. The initial yielding strength is 361.8 MPa, the maximum hardening value of the yield surface is 17.4 MPa, and the ratio of the change in yield strength to the development of plastic strain is 0.7. Three pairs of back stresses are selected. The corresponding parameters of *C*_k_ are 5587.3, 1210.1, and 192.5 MPa, respectively. The material parameters of *γ*_k_ are 89.1, 14.7, and 12.7, respectively. Convergence study was carried out with the dead load applied to the structure. It shows that the convergent stress result at the joint can be obtained when the shell element size is less than 0.25 m. In this study, the element sizes were set as 0.18 m for the arch ribs and 0.015 m for the columns, respectively.

**Figure 10:**
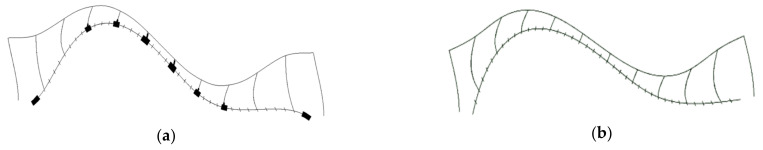

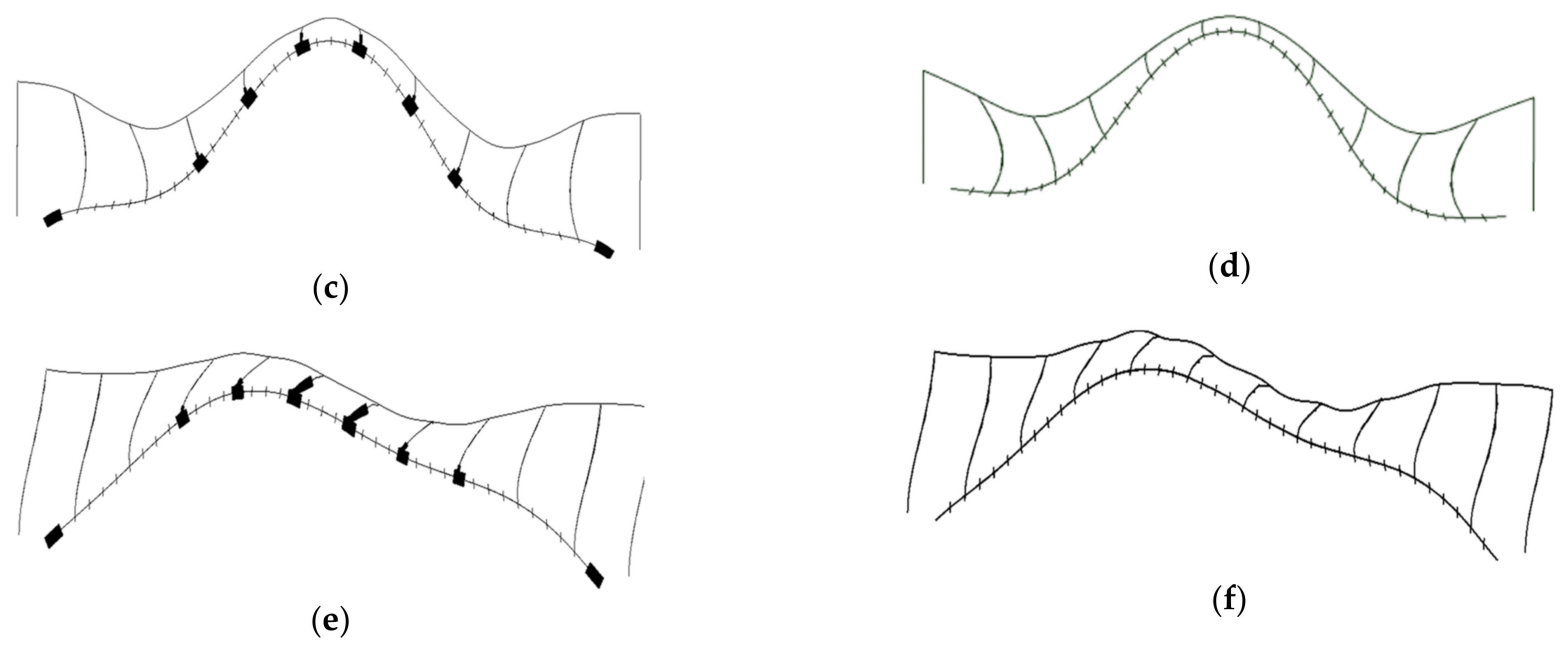
In-plane free-vibration modes of the bridge: (**a**) first-order mode obtained by hybrid element model; (**b**) first-order mode obtained by beam element model; (**c**) second-order mode obtained by hybrid element model; (**d**) second-order mode obtained by beam element model; (**e**) third-order mode obtained by hybrid element model; (**f**) third-order mode obtained by beam element model.

Interaction function of “MPC-beam” provided in the software was used for the connection between different element segments [42]. Figure 10 shows the comparisons of the first three orders of in-plane free vibration modes obtained by the hybrid element model and the full fiber-beam element model. The corresponding natural frequencies of vibration are 0.668, 1.299, and 1.942 Hz, respectively. The natural frequencies and vibration modes obtained from the hybrid element model agree well with the predictions by the fiber-beam element model, indicating the connections between different element segments in the hybrid element model are effective. The hybrid element model was used for further studies in the following sections.

### 4.2. Evaluation of High-Cycle Fatigue Damage during Service Life

HCF damage of the material is cumulatively increasing since the first day of service due to various load excitations. Among these load excitations, the vehicle loads produce the largest fatigue damage. As it is stated in Section 4.1, the arch-column joints in the mid-span position and the arch feet are prone to LCF failure under a seismic event, the HCF damages of the most dangerous arch-column joints (P7 to P10) in the mid-span position are studied. An intelligent weight-in-motion system has been installed in the expressway between Shanghai and Beijing, in which the axle weight, wheelbase, moving velocity, temperature, etc. are recorded at every moment. Long-term monitoring on the traffic flow information was extracted for analysis, in which the information of smaller cars less than 3 tons was ignored. A dimensionless factor *K* is defined, and it is a ratio of the average traffic volume every day in a month and in the whole year

(8) $$K=\frac{Average\;\;\;traffic\;volume\;\;everyday\;\;in\;\;\;a\;\;month}{Average\;\;\;traffic\;volume\;\;everyday\;\;in\;\;\;a\;\;year}$$

**Figure 11:**
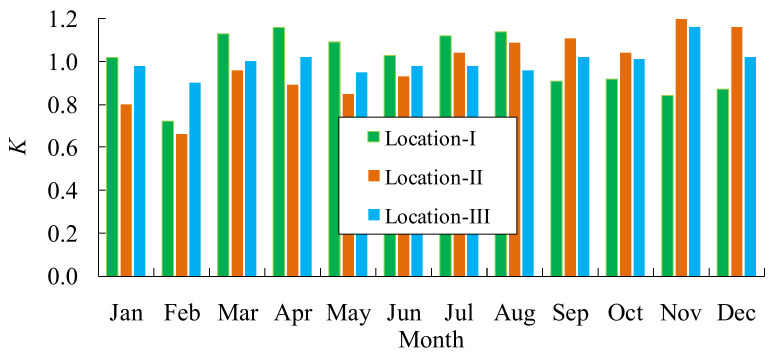
Traffic volume distribution of each month in 2016.

Figure 11 shows the traffic volume distribution in each month in 2016. As it can be seen, the traffic volumes are slightly smaller in January and February because of the Spring Festival holidays. However, the traffic volume is uniformly distributed in the other months.

**Figure 12:**
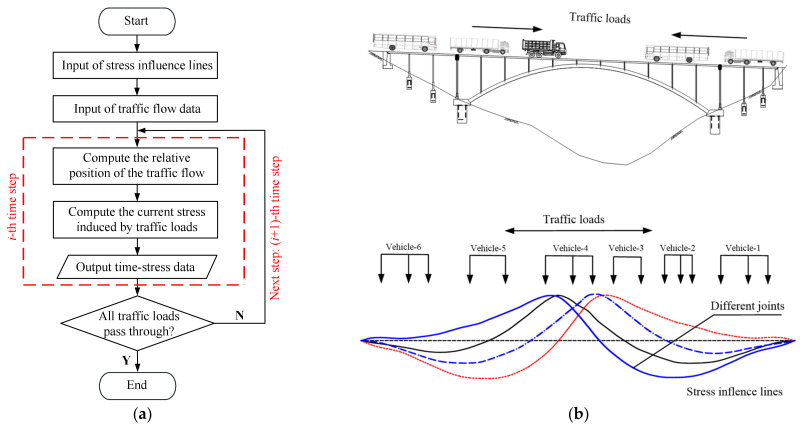
Stress-history analysis of the arch-column joints: (**a**) flowchart of the stress-history analysis; (**b**) movement of the traffic flow on the bridge.

In order to reduce the computational cost, the traffic flow information recorded at Location-II in March was used for the HCF damage accumulation analysis. A constant traffic flow volume was assumed during the whole service life of the bridge. Figure 12 shows the flowchart of the stress-history analysis, in which the traffic loads moved through the bridge in every time increment (Δ*t* = 0.2 s), and the generated stress at the concerned joints was computed. A program coded by FORTRAN language was used for the computation of the stress-history results.

**Figure 13:**
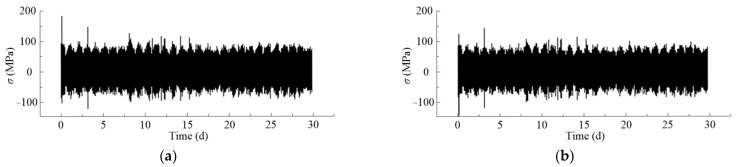
Stress-history curves at the bottom of P8 and P9 columns during service time: (**a**) P8 arch-column joint; (**b**) P9 arch-column joint.

Figure 13 shows the stress-history curves of the arch-column joints P8 and P9. The results show that the stress history changes periodically every day, the larger values appear at daytime, while the relatively smaller values appear at night. As the results were directly obtained by applying the traffic load to the stress influence lines, very large stress responses can be observed when overload vehicles are passing through the bridge. Some trucks in the traffic flow are so overloaded that the corresponding stress responses even exceed 150 MPa.

**Figure 14:**
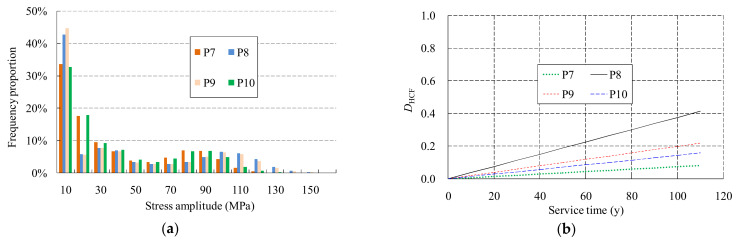
Stress spectrum and fatigue damages accumulated during service: (**a**) stress spectra of the joints; (**b**) HCF damages of the joints.

The rain-flow counting method was used to get the stress spectra at these joints, and the proposed Equation (1) and Miner’s rule were used to obtain the accumulated HCF damages. Figure 14 shows the stress spectra and HCF damages of the joints. As a constant traffic flow and Miner’s rule were adopted during the analysis, linear-increased fatigue damage curves were obtained. As it can be seen, the frequency fraction decreases gradually with the increase of the stress amplitude. The frequency fraction is the largest for the stress amplitude less than 20 MPa, this kind of stress amplitude are generated by smaller trucks. The maximum fatigue damage value at the bottom of P8 column with a service period of 100 years is about 0.4, indicating that no HCF failure occurred during the entire service life.

### 4.3. Evaluation of Low-Cycle Fatigue Damage under Seismic Event

**Figure 15:**
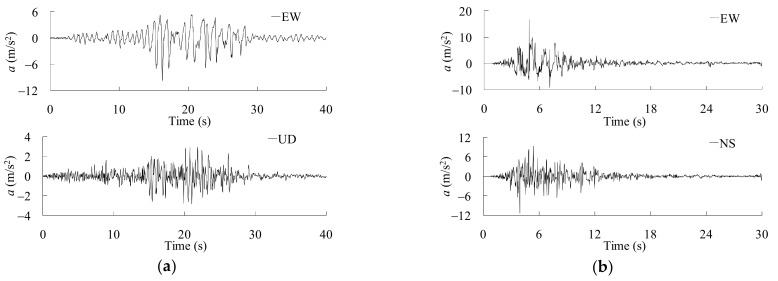
Accelerations of the input earthquakes: (**a**) Chi-chi (1999) earthquake recorded at Sun Moon Lake station; (**b**) Niigata (2004) earthquake recorded at Kawaguchi station.

The nonlinear dynamic response analysis was carried out using the well-known implicit integration algorithm, i.e., the Newmark-*β* algorithm with a constant value of *β* = 1/4. Rayleigh damping was adopted with ratios of 2.0% [43]. Two real recorded strong earthquakes as shown in Figure 15 were selected as the input seismic loads to ensure structural seismic damages.

**Figure 16:**
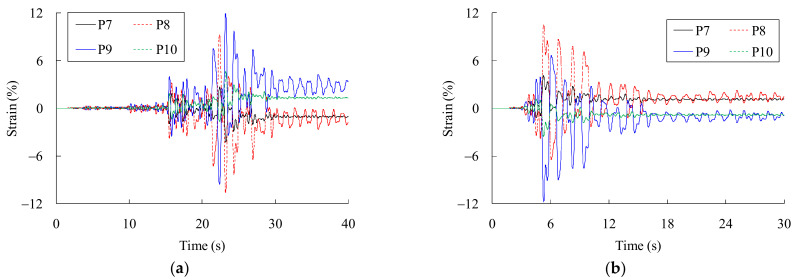
Strain responses of the joints under strong earthquakes: (**a**) Strain responses caused by Chi-chi earthquakes; (**b**) strain responses caused by Niigata earthquakes.

Figure 16 shows the strain responses of the arch-column joints under the seismic events. As the P8 and P9 joints are very close to the mid-span of the bridge, the strain responses there are larger than the other positions. Moreover, the strain responses of P9 and P10 joints are opposite to the responses of P7 and P8 joints, indicating the anti-symmetrical vibration modes of the bridge play an important role in the dynamic response.

**Figure 17:**
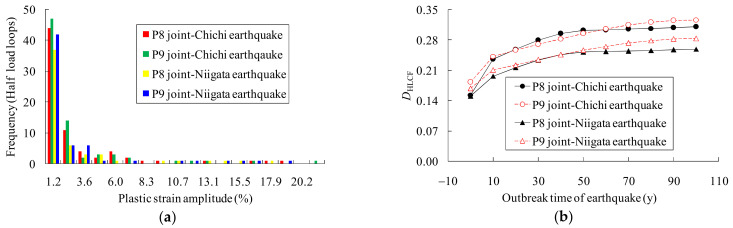
Plastic strain spectra and coupled fatigue damages of the joints: (**a**) plastic strain spectra of the joints; (**b**) HLCF damages of the joints.

Figure 17 shows the plastic strain spectra and the fatigue damage evolutions of the P8 and P9 joints, in which the spectra were obtained based on the dynamic strain responses, and the fatigue damages were computed using Equation (7). The fatigue damage at the beginning of the bridge service is only induced by the seismic event, while it is a combined consideration of the fatigue damages induced by the traffic loads and the seismic loads afterwards. The maximum combined damage index is less than 1.0, indicating no fatigue failure occurred during the whole service life. The HLCF damages of the joints increase rapidly at the beginning, e.g., in the first 40 years of its service life. If the HCF damage accumulation during service is ignored, the fatigue damage in the 100 anniversary of service is about 50% the coupled fatigue damage, which means an unsafe evaluation result is obtained. Therefore, the seismic safety assessment of a steel bridge needs to take into account the outbreak time of the earthquakes. The approach and the proposed formulae in this study can effectively estimate the fatigue damages of an in-service steel structure.

## 5. Conclusions

In this paper, the combined high and low cycle fatigue tests of the low alloy steel Q345 were carried out. Some significant notes were shown experimentally, and prediction formulae for different kinds of fatigue lives were proposed. Finally, the proposed formulae were used for the fatigue damage prediction of an in-service steel arch bridge. Based on the experimental and numerical studies in this paper, some conclusions can be drawn:

(1) The fitting equations were obtained for the HCF and LCF life predictions of the low alloy steel Q345. Noticeably, a new damage index based on the Miner’s rule and Coffin–Manson relation was proposed, which is able to quantitative evaluate the LCF damage caused by variable amplitude loading.
(2) The decrease process of the LCF life can be divided into three stages. The first and third stages are very short, but the fatigue life deteriorates very fast, represented by the relatively larger slope on the figure, while the second stage is relatively long and stable. The HCF lifetime fractions of these three stages are about 20%, 50%, and 30%, respectively. A piecewise formula to predict the HLCF damage was proposed, which is a combined consideration of the damages accumulated during the service history and the damages caused by a seismic event.
(3) The HCF crack initiates from the specimen surface, and the fracture surface is rather flat. The LCF fracture surface is very rugged, and multiple crack sites at different height are observed. The HLCF fracture has the features of both the HCF and LCF fracture surfaces, such as the crack initiation near the surface and the dimples distributed on the fracture surface. Moreover, the HLCF fracture surface is relatively flat near the crack initiation side, and rugged at the other half part.
(4) During the whole service life of the steel bridge, the maximum HCF damage of the joints is about 0.4, indicating no HCF failure caused by the traffic loads occurs.
(5) As the service time goes on, the HLCF damages of the joints increase rapidly, especially in the first 40 years after construction. The seismic safety assessment of a steel bridge needs to account for the worst scenario, e.g., the seismic event occurs at the end of the structural service life. The fatigue damage at the beginning of the service is only induced by the seismic event, and it is about 50% of the damage value at the end of the service life.

The proposed formulae were only based on the material tests of a limited number of specimens, further studies are needed to obtain more experimental data on the critical parameters and to verify the proposed formulae.

## Figures and Tables

**Table 1:** Chemical compositions of the low alloy steel (wt %).

**C**	**Si**	**Mn**	**P**	**S**	**Nb**	**V**	**Ti**
≤0.18%	≤0.55%	0.9~1.70%	≤0.025%	≤0.02%	≤0.06%	≤0.08%	≤0.03%
**Cr**	**Ni**	**Cu**	**Mo**	**N**	**Als**	**Fe**	/
≤0.08%	≤0.50%	≤0.55%	≤0.20%	≤0.012%	≥0.015%	Balance	/

**Table 2:** Test results of the high cycle fatigue.

Specimen No.	Δ*σ*/2 (MPa)	Diameter (mm)	Fatigue Life	Average Life
HCF-1-1	±200	6.51	1,556,500	1,672,467 (0.057)
HCF-1-2	±200	6.48	1,671,700	
HCF-1-3	±200	6.48	1,789,200	
HCF-2-1	±220	6.49	885,100	768,565 (0.108)
HCF-2-2	±220	6.51	725,945	
HCF-2-3	±220	6.52	694,650	
HCF-3-1	±230	6.47	193,000	167,313 (0.115)
HCF-3-2	±230	6.45	162,400	
HCF-3-3	±230	6.49	146,540	
HCF-4-1	±250	6.52	34,700	39,600 (0.147)
HCF-4-2	±250	6.48	47,800	
HCF-4-3	±250	6.48	36,300	

**Table 3:** Test results of the low cycle fatigue.

Specimen No.	Δ*ε*/2 (%)	Diameter (mm)	Fatigue Life	Average Life
LCF-1-1	±1.0	6.50	364	405.0 (0.072)
LCF-1-2	±1.0	6.49	430	
LCF-1-3	±1.0	6.51	421	
LCF-2-1	±1.5	6.49	276	304.7 (0.069)
LCF-2-2	±1.5	6.51	312	
LCF-2-3	±1.5	6.49	326	
LCF-3-1	±2.0	6.49	214	194.7 (0.089)
LCF-3-2	±2.0	6.47	198	
LCF-3-3	±2.0	6.52	172	
LCF-4-1	±2.5	6.51	128	139.0 (0.062)
LCF-4-2	±2.5	6.51	140	
LCF-4-3	±2.5	6.51	149	
LCF-5-1	±3.0	6.50	71	77.7 (0.179)
LCF-5-2	±3.0	6.47	65	
LCF-5-3	±3.0	6.48	97	

**Table 4:** Test results of the combined high and low cycle fatigue.

Specimen No.	Diameter (mm)	Stage I: Load Loops	Stage II: Fatigue Life	Average Life
HLCF-1-1	6.52	0.1*N_Hf_*	58	87.7 (0.270)
HLCF-1-2	6.50		89	
HLCF-1-3	6.51		116	
HLCF-2-1	6.51	0.2*N_Hf_*	100	108.7 (0.211)
HLCF-2-2	6.51		86	
HLCF-2-3	6.48		140	
HLCF-3-1	6.52	0.3*N_Hf_*	127	90.3 (0.321)
HLCF-3-2	6.50		56	
HLCF-3-3	6.50		88	
HLCF-4-1	6.50	0.4*N_Hf_*	79	83.3 (0.040)
HLCF-4-2	6.51		84	
HLCF-4-3	6.50		87	
HLCF-5-1	6.50	0.5*N_Hf_*	131	104.7 (0.244)
HLCF-5-2	6.52		70	
HLCF-5-3	6.51		113	
HLCF-6-1	6.50	0.6*N_Hf_*	90	91.0 (0.032)
HLCF-6-2	6.50		95	
HLCF-6-3	6.50		88	
HLCF-7-1	6.52	0.7*N_Hf_*	42	94.3 (0.392)
HLCF-7-2	6.51		122	
HLCF-7-3	6.51		119	
HLCF-8-1	6.48	0.8*N_Hf_*	70	64.0 (0.111)
HLCF-8-2	6.50		54	
HLCF-8-3	6.52		68	
HLCF-9-1	6.50	0.9*N_Hf_*	38	37.5 (0.013)
HLCF-9-2	6.51		/ ^1^	
HLCF-9-3	6.50		37	

^1^ Specimen failure occurred in Stage I.

## Data Availability

Data is contained within the article.

## Nomenclature

*a*	Acceleration of the input earthquake wave
*C*	Material parameter in the Coffin–Manson relation
*C*_k_	Initial modulus of the kinematic hardening
*D*_HCF_	HCF damage index
*D*_LCF_	LCF damage index
*D*_HLCF_	HLCF damage index
*K*	Ratio of the average traffic volume every day in a month and in the whole year
*k*	HCF life fraction
*k’*	Constant material parameter in the Coffin–Manson relation
*N_Hf_*	HCF life
*N_Lf_*	LCF life
*N_HLf_*	HLCF life
*N_Lfi_*	LCF life corresponding to the *i*-th level amplitude strain load
*n*	Number of the applied load cycles
*n_i_*	Number of the applied load cycles with the *i*-th level amplitude
*R*	Strain ratio of the fatigue load
Δ*t*	Time increment during the stress-history analysis
*β*	Parameter in the Newmark-*β* algorithm
*γ*	Reduction coefficient considering the effect of the HCF pre-damage
*γ_k_*	Ratio of the kinematic hardening modulus change to the plastic strain
Δ*σ*	Full stress load amplitude
Δ*ε*	Full strain load amplitude
Δ*σ*/2	Stress load amplitude
Δ*ε*/2	Strain load amplitude
Δ*ε_pi_*	Plastic strain amplitude in the *i*-th half load loop
Δ*ε_p_*/2	Plastic strain load amplitude
